# Stress-Induced Intensification of Deoxyshikonin Production in *Rindera graeca* Hairy Root Cultures with Ester-Based Scaffolds

**DOI:** 10.3390/plants11243462

**Published:** 2022-12-10

**Authors:** Kamil Wierzchowski, Mateusz Kawka, Michał Wrzecionek, Julia Urbanek, Agnieszka Pietrosiuk, Katarzyna Sykłowska-Baranek, Agnieszka Gadomska-Gajadhur, Maciej Pilarek

**Affiliations:** 1 Faculty of Chemical and Process Engineering, Warsaw University of Technology, Waryńskiego 1, 00-645 Warsaw, Poland; 2 Department of Biology and Pharmacognosy, Faculty of Pharmacy, Medical University of Warsaw, Banacha 1, 02-097 Warsaw, Poland; 3 Faculty of Chemistry, Warsaw University of Technology, Noakowskiego 3, 00-664 Warsaw, Poland

**Keywords:** hairy roots, *Rindera graeca*, stress-inducing, deoxyshikonin, polymerized organic ester-based constructs

## Abstract

In vitro plant cell and tissue culture systems allow for controlling a wide range of culture environmental factors selectively influencing biomass growth and the yield of secondary metabolites. Among the most efficient methods, complex supplementation of the culture medium with elicitors, precursors, and other functional substances may significantly enhance valuable metabolite productivity through a stress induction mechanism. In the search for novel techniques in plant experimental biotechnology, the goal of the study was to evaluate stress-inducing properties of novel biodegradable ester-based scaffolds made of poly(glycerol sebacate) (PGS) and poly(lactic acid) (PLA) influencing on the growth and deoxyshikonin productivity of *Rindera graeca* hairy roots immobilized on the experimental constructs. *Rindera graeca* hairy roots were maintained under the dark condition for 28 days in three independent systems, i.e., (i) non-immobilized biomass (a reference system), (ii) biomass immobilized on PGS scaffolds, and (iii) biomass immobilized on PLA scaffolds. The stress-inducing properties of the applied polymerized esters selectively impacted *R. graeca* hairy roots. The PGS scaffolds caused the production of deoxyshikonin, which does not occur in other culture systems, and PLA promoted biomass proliferation by doubling its increase compared to the reference system.

## 1. Introduction

The biotechnological approaches to in vitro plant cultures provide significant advantages over conventional methods, overcoming limitations imposed by plant biology, environmental factors, or legal issues [1]. Moreover, their applicability covers the possibility of harnessing plants’ unique biosynthetic potentials using renewable feedstock to produce hardly available compounds, such as secondary plant metabolites [2].

The engineering of in vitro systems for plant cell and tissue bioprocessing allows for the precise control of the culture environment, influencing biomass growth and the yielding of secondary metabolites [3]. The secondary metabolites play a key role in defense reaction and adaptation to the constantly changing environment [4]. They are biosynthesized de novo upon various biotic and abiotic stress factors. This phenomenon was applied in biotechnology to stimulate the production of plant-derived biocompounds [5]. Among other techniques, the immobilization of in vitro plant biomass demonstrated one of the most significant results in the productivity enhancement of valuable metabolites [6]. In the case of hairy roots, there is a lack of extensive research on the immobilization of such plant organs, despite their unique and unequivocally high biosynthetic potential. Due to metabolic pathways specific to the roots’ tissues and the activity of endogenous phytohormones, hairy roots are characterized by enhanced growth kinetics and intensified production of secondary metabolites with altered phytochemical profiles [7]. The ability of in vitro cultures to maintain hairy roots with restricted control of environmental parameters generates a synergistic advantage in the redirection of plant tissue metabolism.

Previously, the immobilization of hairy roots on organosilica aerogels, i.e., porous solid biomaterials, was introduced as a promising bioengineering technique for enhanced growth efficiency and the secondary metabolites yield in cultures of *R. graeca* biomass [8]. Moreover, it has been discussed that differences in the physicochemical properties of applied biomaterial-based constructs could be the main factor influencing the metabolic response of cultured biomass. The matched affinity of the biomaterial toward specific secondary metabolites resulted in simultaneous immobilization and in situ adsorption of the desired product with its accumulation in the additional solid phase, leading to enhanced productivity [9].

In the available literature, only a few papers consider applying polymer materials for hairy root immobilization [8,10,11,12,13]. The polymer material used in the hairy root cultivation was applied for the first time in the cultures of *Azadirachta indica* root biomass supported on the polyurethane foam (PUF) [10]. The PUF scaffolds were also used for the cultures of *Catharanthus roseus* hairy roots [11] and *Rindera graeca* hairy roots [8]. The other polymer applied in the hairy root biomass immobilization was polypropylene (PP), which was used for supporting *Plumbago rosea* hairy roots [12] and *Azadirachta indica* root biomass [13]. Considering the demand for plant secondary metabolites, which have very complex chemical structures, novel biomaterials with properties tailored to their intended functionality are strongly needed, and their applicability needs to be tested. In this case, we were considering the application of poly(lactic acid) (PLA) and poly(glycerol sebacate) (PGS) and their stress-inducing properties for deoxyshikonin production in *R. graeca* hairy roots.

The PLA is probably the most researched and utilized biodegradable and renewable aliphatic polyester [14]. The water contact angle for PLA is ca. 70–80 degrees, which indicates its relatively hydrophilic nature. This property causes low cell affinity, which could be the reason for the inflammatory response of the PLA-based constructs after their direct contact with biomass [15]. The degradation rate of PLA is relatively low, allowing such biopolymers to have a long lifetime in in vivo applications. In some cases, the lifetime of PLA scaffolds could reach up to 3–5 years [16]. Young Modulus of PLA materials could be achieved in the range of 1 to 4 GPa, depending on the synthesis methodology [14]. Currently, PLA has found many practical applications in different fields [17], e.g., textiles, packaging materials, and composites. However, from the bioengineering point of view, the most attractive application of PLA materials in in vivo systems is the development of drug-delivery systems in new forms and new tissue engineering materials [18,19,20,21].

The PGS was originally synthesized in 2002 and immediately recommended as a flexible biomaterial with many possible modifications of its properties [22,23]. Despite its wide application in tissue engineering [24,25,26], PGS is a much less known applicable material than PLA. Furthermore, new applications of PGS are still being discovered due to the fact that its properties strongly depend on its structure, which directly results from the synthesis methodology. The PGS has a high potential to become a “tailor-made” material [27]. It has rubber-like mechanical properties with a Young modulus ranging from 0.03 to 1.2 Mpa [28]. Its many applications are focused on the medical industry, e.g., soft tissue bioengineering [29] and various drug delivery systems [30].

Among the secondary plant metabolites, much attention is focused on shikonin derivatives, which belong to the naphthoquinones group. These lipophilic red pigments were thoroughly investigated due to their anti-cancer and anti-inflammatory properties, being potentially valuable products for the pharmaceutical industry [31,32,33].

Our original concept presented in the current study is the evaluation of novel techniques in plant experimental biotechnology. The primary goal of the study was to evaluate stress-inducing properties of novel biodegradable ester-based scaffolds made of poly(glycerol sebacate) (PGS) and poly(lactic acid) (PLA) influencing on the growth and deoxyshikonin productivity of *R. graeca* hairy roots immobilized on the experimental constructs. Based on our previous research [8,34,35,36,37], we selected the hairy roots of *Rindera graeca* (*Boraginaceae*), which are known for the presence of naphthoquinone derivatives in their phytochemical profile, for further investigation. Poly(glycerol sebacate) and poly(lactic acid) were selected due to their porous structure, biocompatibility, acceptable wettability, and degradability to monomers which are non-toxic for plant tissues. The presented methodology introduces the application of novel synthetic biopolymers in plant biomass in vitro culture systems for the purpose of secondary metabolites manufacturing.

## 2. Results

### 2.1. Polymerized Organic Ester-Based Constructs Characterization

**Figure 1:**
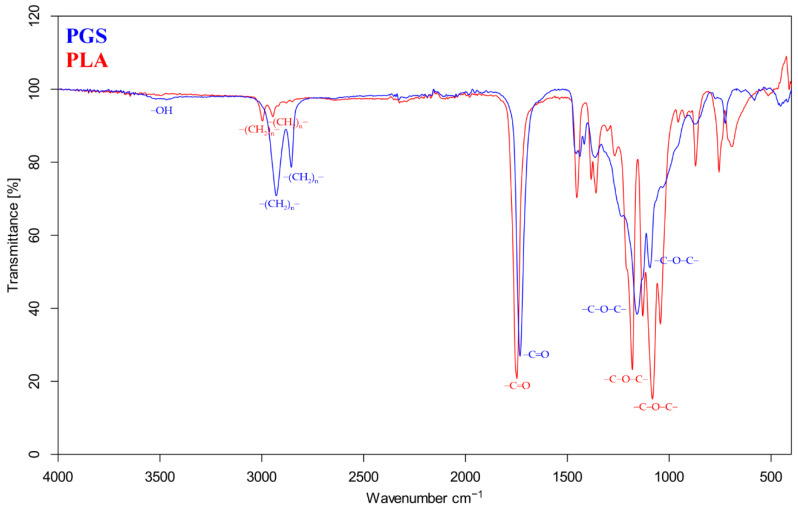
FT-IR spectra for studied materials: PGS—blue line, PLA—red line. The peaks corresponding to individual chemical groups are marked with the following symbols: −OH—hydroxy group, -−(CH_2_)_n_−—methylene groups from aliphatic chain, −C=O—carbonyl group from ester carbonyl moiety, −C−O−C− ether group from ester carbonyl moiety.

The chemical structure of both synthesized organic-ester-based materials further applied for scaffold preparations, i.e., PGS and PLA, was slightly different, confirmed by FT-IR spectroscopy (Figure 1).

As it can be easily recognized based on the data presented in Figure 1, characteristic bands for each polyester substrate exist. In the case of the PLA constructs, there are two bands (i.e., 2997 and 2855 cm^−1^) specific for the aliphatic chain and three bands (i.e., 1748 cm^−1^ for −C=O group from ester carbonyl moiety and 1184 cm^−1^, 1093 cm^−1^ for −C−O−C− group from ester carbonyl moiety) specific for the ester carbonyl group. There are also bands (i.e., 2929 and 2855 cm^−1^) in PGS spectra for the aliphatic chain but with stronger intensity due to the longer acid chain (i.e., PGS). The ester carbonyl characteristic bands are slightly shifted (i.e., 1732 cm^−1^ for −C=O group from ester carbonyl moiety and 1161 cm^−1^, 1106 cm^−1^ for −C−O−C− group from ester carbonyl moiety) to a lower wavelength. Moreover, the wide band characteristic for hydroxy groups (i.e., 3501 cm^−1^) could testify to the presence of unreacted acid and hydroxy groups. However, the amount of these groups is inconsiderable. The data presented in FT-IR spectra are consistent with the results of other research groups for both studied polyesters, i.e., PLA [38] and PGS [39].

**Figure 2:**
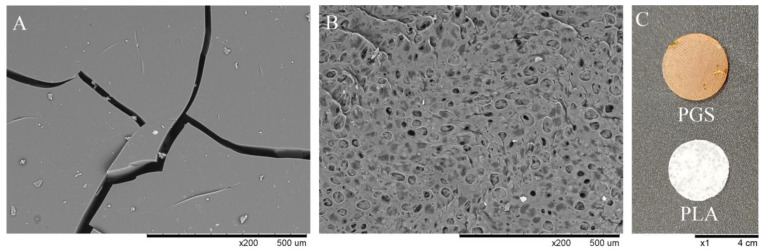
SEM micrographs of materials applied for scaffolds: (**A**) non-porous PGS with many deep cracks in the whole volume of the sample, (**B**) porous PLA with approx. 50–75 μm pores, and (**C**) macroscopic view of PGS and PLA scaffolds.

Scanning electron microscope (SEM) imaging of PGS and PLA samples is presented in Figure 2A,B. In SEM micrographs, PGS constructs have many deep cracks throughout the whole volume of the sample, but the solid tiles of PGS were non-porous (Figure 2A). In the case of PLA constructs, it has a highly porous structure, where the diameter of pores is approximately 50–75 μm (Figure 2B).

The macroscopic view of PGS and PLA scaffolds is presented in Figure 2C. Both materials have round shapes and are 4 cm in diameter, but their thickness is different and is 3 mm for the PGS scaffold and 0.5 mm for the PLA scaffold. Moreover, they significantly varied in morphology and flexibility. The PGS scaffolds were highly flexible and elastic (i.e., rubber-like), but PLA scaffolds were relatively inflexible. The crosslinking degree of the PGS scaffold was 90%, measured as gel content.

**Figure 3:**
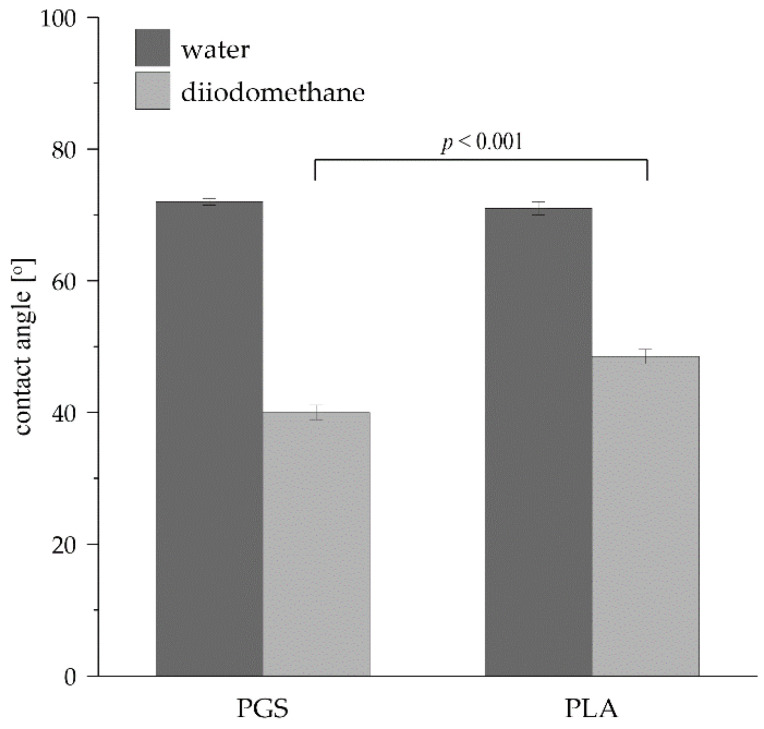
The values of water and diiodomethane contact angles measured for PGS and PLA applied for scaffold preparation. The *p* values over brackets were determined according to the student’s *t*-test.

The values of water and diiodomethane contact angles measured for PGS and PLA are presented in Figure 3. Water contact angle values were similar for both materials, equaling 71–72°, which characterizes the materials with hydrophilic properties. The water contact angle values for both constructs are similar to those obtained by other researchers [19,27]. In the case of diiodomethane contact angle values, there are slight differences between these values for studied materials: the value noticed for PLA was equal to 48.5° vs. the value measured for PGS, which was equal to 40.0°. The lower value of the diiodomethane contact angle for PGS-based constructs indicates a better affinity for non-polar substances than PLA-based constructs.

The surface free energy values (SFE), which describe the excess energy on the material surface per material mass, and the density values are presented in Table 1. A higher value of SFE was observed for PLA-based constructs (15.2 mJ m^−2^), and it was almost 8 times higher than the SFE value noticed for PGS-based constructs (1.74 mJ m^−2^). Higher values of SFE indicate the more adhesive surface of PLA scaffolds than the surface of PGS scaffolds. In the case of density values, the higher value was reported for PGS-based constructs, equaling 1.21 g cm^−3^. While the density value noticed for the PLA-based construct was almost 10 times lower than PGS one, equaling 0.127 g cm^−3^. If we compare the density of PLA and PGS scaffolds to water density at 24 °C, which may be approximately equal to the density of the culture medium (0.997 g cm^−3^), the PLA-based constructs float on the culture medium surface, and the PGS-based constructs sink in the culture medium. The SFE and density values for PLA constructs are similar to values of these parameters obtained by other researchers [40]. In the case of PGS constructs, there is no data on the SFE values in the literature. However, the density values are consistent with the literature data [27].

**Figure 4:**
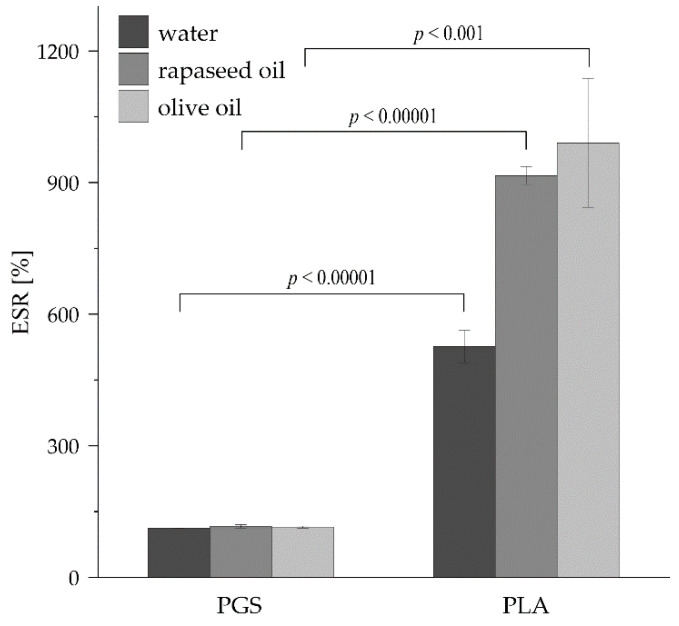
The ESR values of water, rapeseed oil, and olive oil estimated for studied constructs made of PGS and PLA. The *p* values over brackets were determined according to the student’s *t*-test.

Equilibrium swelling ratio (ESR) values describing the swelling properties of the studied materials were measured for three different liquids, i.e., water, rapeseed oil, and olive oil, and the corresponding values are compared in Figure 4. The ESR values observed for the PGS-based construct were almost the same for all tested liquids, equaling between 112% and 116%. This value is consistent with the previously published by other research group results [27]. However, the values of ESR for the constructs made of PLA were significantly higher compared to the PGS-based ones. The values characterizing rapeseed oil and olive oil absorption by PLA-based constructs (915% and 990%, respectively) were almost two times higher than the value of ESR for water (526%). The values of water ESR for PLA are similar to the values obtained by other researchers [41]. The ESR values for the water show the culture medium capacity of studied materials, and rapeseed oil and olive oil ESR values may be related to deoxyshikonin capacity. In both cases, the PLA-based constructs exhibited much better properties than PGS-based ones.

### 2.2. Biomass Proliferation and Deoxyshikonin Production

**Figure 5:**
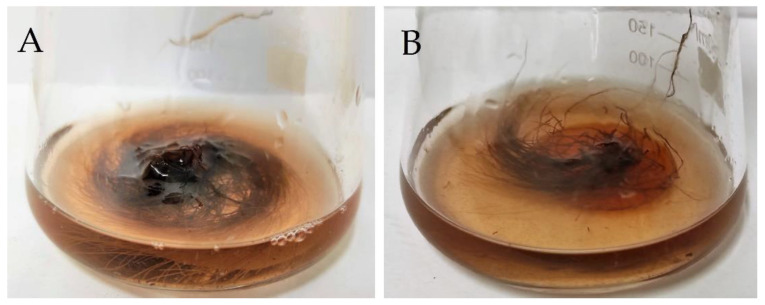
Morphology of *R. graeca* hairy roots immobilized on investigated organic-ester-based constructs: (**A**) hairy roots elevated to the gaseous phase by PLA scaffold, and (**B**) hairy roots fully submerged inside the liquid phase on PGS scaffold (after 28 days of in vitro culture).

Both investigated organic ester-based constructs were well-suited to the culture system dimensions (Figure 5) and were resistant to shear forces generated by mixing conditions, allowing for a scheduled 28-day in vitro culture of *R. graeca* hairy roots.

**Figure 6:**
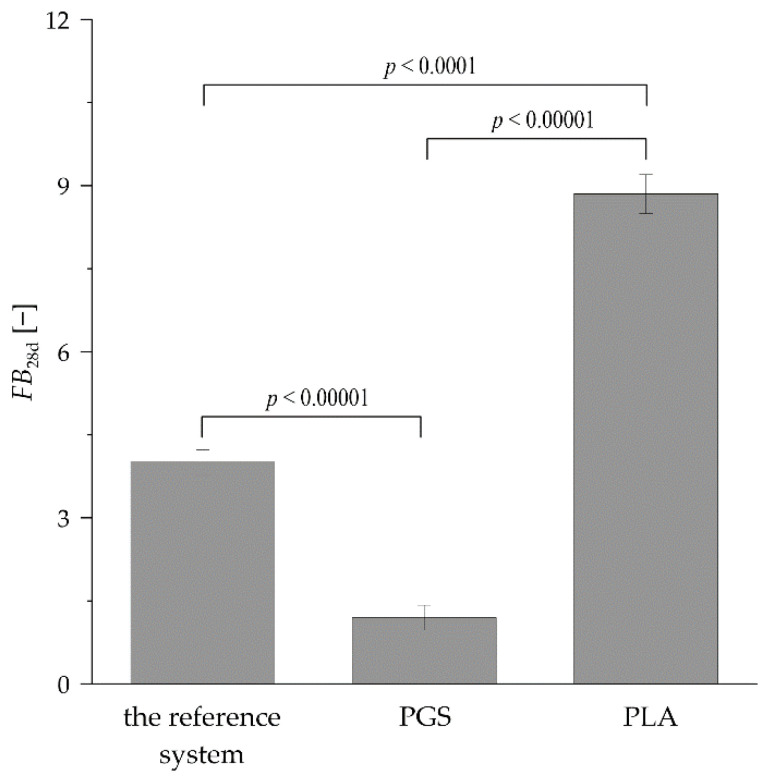
FB_28d_ values characterize in vitro proliferation of *R. graeca* hairy roots cultured in the reference system and on organic ester-based scaffolds made of PGS and PLA. The *p* values over brackets were determined according to the ANOVA test.

The values of fresh biomass increase (FB_28d_) noted for *R. graeca* hairy roots proliferated on biodegradable polymers after 28 days are compared in Figure 6. The PLA-based culture system had the highest FB_28d_ value (8.85), followed by the reference system (4.01) and the PGS-based culture system (1.12).

**Figure 7:**
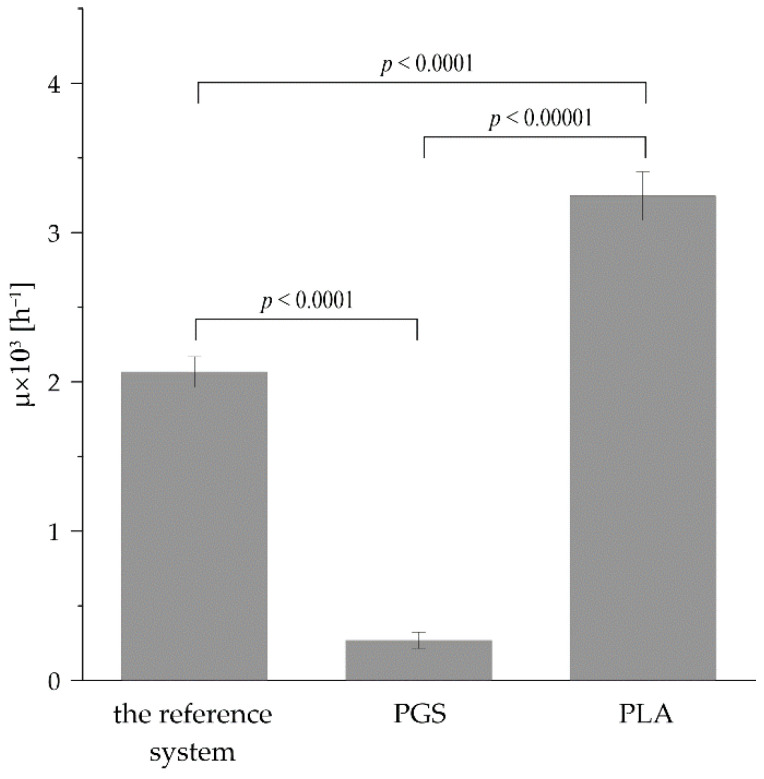
The values of µ characterizing in vitro cultures of *R. graeca* hairy roots proliferated in the reference system and systems supported with PGS- and PLA-based scaffolds. The *p* values over brackets were determined according to the ANOVA test.

The values of specific growth rate (μ) noticed for *R. graeca* hairy root cultures supported with applied organic-ester-based constructs are presented in Figure 7. The PLA-based culture system had the highest μ value (3.245 × 10^−3^ h^−1^), followed by the reference system (2.065 × 10^−3^ h^−1^) and the PGS-based culture system (0.269 × 10^−3^ h^−1^).

Values of m_n_ characterizing deoxyshikonin weight produced in the culture system and values of Y_P/X_ characterizing the productivity of deoxyshikonin noted for three in vitro culture systems are compared in Table 2. In the case of the reference culture non-supported with any construct and the culture supported with the PLA-based construct, the values of m_n_ and Y_P/X_ equaled 0 µg and 0 µg g_DW_^−1^, respectively. This was caused by the level of deoxyshikonin under the detection threshold characterizing such culture systems. However, in the case of the hairy root cultures supported with the PGS-based scaffold, the values of m_n_ and Y_P/X_ were equal to 157.4 µg and 1983 µg g_DW_^−1^, respectively.

## 3. Discussion

Previously investigated solutions for metabolic engineering via biomass immobilization or bioproduct in situ adsorption performed in plant in vitro culture systems are presented in Table 3. The joint objective presented in all cited studies was the intensification of secondary metabolite biosynthesis. In most of the articles listed in Table 3, Y_P/X_ and FB_28d_ were calculated based on the equations from Section 4.5, as the values of these parameters were not given directly in the papers.

Due to the differences in species-specific phytochemical profiles of the investigated plants and in vitro maintained plant biomass, conclusions drawn from the study analyses are not directly comparable. One thing is clear: among the examples mentioned above, the highest secondary metabolite yield (i.e., 11,076 µg g_DW_^−1^) was noted for the culture system supported with DIAION HP-20 polystyrene resin for in vitro cultures of *Arachis hypogaea* hairy roots producing stilbenes, i.e., resveratrol and its derivatives [44]. Developed protocol of the resin-based extraction of stilbenes from the culture medium significantly simplified the whole process, allowing for high efficiency with less amount of the organic solvent. As it turns out, polyurethane foam (PUF) was frequently applied as the supporting construct for in vitro proliferated hairy roots. Due to the PUF having a lightweight, resilient, and porous structure with a high affinity toward non-ionic lipophilic compounds, efficient production of tetranotriterpenoids [13] ajmalicine [11] and naphthoquinones [8] in hairy roots was reported with Y_P/X_ productivity of 6400 µg g_DW_^−1^, 1130 µg g_DW_^−1^ and 653 µg g_DW_^−1^, respectively. Establishing the PUF-supported in vitro culture system significantly enhanced the possibility of biomass contact with the gaseous (i.e., air-based) phase inside the culture flask, in contrary to typical culture systems in which totally submerged biomass is maintained.

Moreover, the results of some experiments clearly demonstrated the bifunctionality of the investigated PUF-based platforms. Nowak et al. [8] reported that PUF was the main place of the culture system for naphthoquinone accumulation allowing for simultaneous in situ extraction of the desired secondary metabolite. Additionally, the authors observed similar results for the increased naphthoquinone accumulation in constructs made of MTMS-based aerogel supporting in vitro cultures of *R. graeca* hairy roots [8]. Amberlite ion-exchange resins XAD-4 and XAD-7 are also noteworthy with their purpose of diterpenoids and thiophenes recovery improvement from the systems of in vitro cultures of *Salvia miltiorrhiza* [42] and *Tagetes patula* [43], respectively.

Neither PLA nor PGS was applied, neither to immobilize in vitro maintained hairy roots nor to investigate/enhance shikonin derivative production—deoxyshikonin. Thus, results obtained in the current study demonstrated the successful application of two novel organic-ester-based polymerized materials for in vitro bioprocessing of plant biomass, with various results regarding biomass growth and secondary metabolites production. The experimental data presented in Table 2 revealed that the production of deoxyshikonin has occurred only by *R. graeca* hairy roots proliferated on PGS-based constructs. For non-immobilized biomass and hairy roots cultured on PLA-based constructs, the level of deoxyshikonin was lower than the detection limit. In vivo biosynthesis of secondary metabolites, e.g., deoxyshikonin, by plants in their natural environment can be induced by a range of stress factors, such as temperature, pH level, microbial, fungal, or insect infection [45,46]. We assume that in the case of the culture system supported with PGS-based constructs presented in our studies, the deoxyshikonin production could be induced by the occurrence of stress factors, which do not occur in other culture systems, i.e., PLA-supported and reference systems. PGS constructs, the non-porous and 6-thicker than PLA ones, could limit the access of hairy roots to the medium, which suppressed their proliferation as a result of water and nutrient element deprivation (Figure 6). The observed induction of deoxyshikonin production could be a result of the initiation of multiple signaling pathways, leading to the production of this phytoalexin that is to allow cells to adapt to detrimental conditions [5]. Other hypothetical stress factors that could affect the secondary metabolism and inhibit the growth of *R. graeca* hairy roots could be products of the degradation of PGS constructs. On the other hand, the sebacate monomers in PGS molecules have a significantly longer aliphatic chain than lactate monomers in PLA (see Figure 1, wavenumbers 2929 and 2855 cm^−1^). The PGS aliphatic chain contains eight carbon atoms, while PLA aliphatic chain contains only one carbon atom. This could be the strong factor influencing interactions between lipophilic deoxyshikonin and PGS, eventually leading to observed bioproduct accumulation in PGS-based constructs. Moreover, the low value of the diiodomethane contact angle has proven good properties of deoxyshikonin accumulation for PGS scaffolds (Figure 3). Furthermore, the crosslinked PGS has wider spaces between ester moieties than crosslinked PLA, which may have influenced the interaction between scaffolds and hairy root biomass. The length of the aliphatic chain for crosslinked PGS is quite similar to the aliphatic chain length in PUF, which was successfully applied for naphthoquinones production from the hairy root biomass [8,47]. Moreover, both materials, i.e., PGS and PUF, inhibited the growth of hairy root biomass. It is possible that these materials influence hairy root biomass in a similar way.

Another difference between investigated materials, PGS and PLA, was the high porosity of PLA-based constructs, which allows for higher accessibility of the culture medium compounds compared to non-porous PGS-based constructs (see Figure 2.). The high ESR values of PLA scaffolds (see Figure 4) are also related to their porosity. Moreover, pores provide a suitable environment for the attachment of the hairy roots on the bottom PLA-based constructs surface, promoting plant biomass proliferation. Another parameter that is correlated with hairy root proliferation is SFE. The PLA scaffolds were characterized by higher SFE values than PGS scaffolds (see Table 1), which indicates the better adhesive properties of the surface of PLA and may cause the increment of the transgenic roots’ proliferation. The next difference between PGS and PLA was enhanced contact with the gaseous phase of the culture system in the case of the latter biomaterial (Figure 5). PLA-based constructs, with their low density (i.e., 0.127 g cm^−3^), caused the elevation of roots to the surface of the aqueous phase, increasing the availability of oxygen for gas exchange. In contrast, due to significantly higher density (i.e., 1.121 g cm^−3^) than water density, biomass cultured on PGS-based constructs was fully submerged inside the liquid phase of the culture medium. It is well-documented in the literature that the lack of availability of respiration gases, i.e., O_2_ and CO_2_, can be the main limiting factor for plant biomass growth in in vitro culture systems [48,49]. Reflection of this phenomenon was probably also observed in the current study as the best results of the biomass growth were found for *R. graeca* hairy roots proliferated on PLA-based constructs.

Based on the results of previously published experiments, the data presented in the current study should be referred to as the results of *R. graeca* hairy root proliferation and deoxyshikonin production observed in the culture systems supported with PUF or constructs made of MTMS-aerogel (please see Table 3). Direct comparison among all four materials (i.e., PGS, PLA, MTMS-based aerogel, and PUF) supporting in vitro culture systems for *R. graeca* hairy roots clearly show the highest growth promoting properties of PLA and most efficient secondary metabolism intensification by PGS. However, further in-depth studies are required to elucidate the mechanism of deoxyshikonin biosynthesis induction in *R. graeca* hairy roots cultivated on polymerized organic-ester-based biomaterials that could lead to enhanced productivity of the proposed culture systems.

## 4. Materials and Methods

### 4.1. Biomaterials Synthesis and Their Basic Characterization

#### 4.1.1. Poly(Glycerol Sebacate) Substrate Preparation

The universal method of producing substrates from glycerol polyester comprises three stages: resin synthesis, crosslinking and rinsing of biomaterial from non-cross-linked fractions.

The resin syntheses were performed in a three-neck round bottom flask equipped with a temperature sensor, magnetic stirrer (RCT basic, IKA, Königswinter, Germany), and Dean-Stark apparatus. 0.40 mol of anhydrous glycerin (Sigma-Aldrich, Burlington, MA, USA) and 0.80 mol of dimethyl sebacate (Sigma Aldrich, Burlington, MA, USA) were weighed into the flask. The reaction was carried out for 30 h at the temperature of 160 °C, and the stirrer rotation was set at 300 rpm.

For the resin to be formed into discs with a diameter of 40 mm, it was poured into polytetrafluoroethylene (PTFE) molds and then immediately crosslinked in a SUP-100W forced air circulation dryer (Wamed, Warsaw, Poland) for 72 h at the temperature of 150 °C. The molds were then cooled to approximately 45 °C, and the prepared discs of biomaterial were removed.

The discs were rinsed twice in DMSO (Sigma Aldrich, Burlington, MA, USA) and then thrice in demineralized water, with the solvent exchanging every 24 h. A glass vessel with discs submerged in solvent was sealed with a glass cap to prevent solvent evaporation. Each time 50 cm^3^ of solvent per disc was used. Baths were carried out on Promax 1020 shaker (Heidolph, Schwabach, Germany) equipped with an Incubator 1000 thermostatic module (Heidolph, Schwabach, Germany) at 25 °C and 100 rpm. The biomaterial discs were then frozen and lyophilized at 0.05 mbar for 96 h and stored until sterilization.

#### 4.1.2. Poly(Lactic Acid) Substrate Preparation

Firstly, a 6% solution of poly(lactic acid) (PLA, 85 kDa, PDI = 2, NatureWorks, Plymouth, MN, USA) in 25 cm^3^ of chloroform (Sigma Aldrich, Burlington, MA, USA) was prepared. The flask was closed and tightly secured with parafilm, and the polymer was left for dissolution for 24 h. After that, 2.38 g of polyvinylpyrrolidone (Sigma Aldrich, Burlington, MA, USA) was added, and the mixture was stirred for another 24 h on a magnetic stirrer (RCT basic, IKA, Königswinter, Germany). After that, the scaffolding mixture was poured onto a cleaned glass plate. The liquid was spread over the plate using a special Elcometer 3580 application knife (Elcometer, Manchester, UK). The glass plate was placed in a ceramic cuvette with methyl alcohol until the mixture coagulation occurred (approximately 20 min). For another 24 h, the substrate was washed in the methanol (Sigma Aldrich, Burlington, MA, USA) bath to rinse the blowing agent from its structure. The substrate was finally air-dried at ambient temperature. After that, the substrate was cut into 40 mm diameter circles.

#### 4.1.3. Sterilization Procedure

All the constructs made of tested biomaterial were packed in vacuum bags intended for food storage. The substrates were sterilized by irradiation with γ-rays. A dose of 25 kGy was applied for 1 min.

#### 4.1.4. FTIR Spectroscopy

Infrared spectra were obtained with the ALPHA II Platinum ATR spectrometer (Bruker, Billerica, MA, USA). For each sample, 32 scans in the range 400–4000 cm^−1^ were performed and averaged.

#### 4.1.5. SEM Imaging

The morphology of the scaffolds was analyzed by SEM imaging on a TM100 electron microscope (Hitachi, Tokyo, Japan) with different magnifications. Before imaging, each sample was covered with an approximately 7-10 nm gold layer using K550X Sputter Coater (Emitech, Bytom, Poland).

#### 4.1.6. Surface Free Energy and Wettability

Water and diiodomethane contact angles on investigated polymer discs were measured using the OCA Physics equipment (Dataphysics Instruments, Filderstadt, Germany). For each solvent, five measurements were made, with the results averaged. Surface free energy (SFE) was calculated in accordance with Owens, Wendt, Rabel, and Kaelble’s method.

#### 4.1.7. Density Estimation

The density of polymer discs was estimated for five samples of each disc by weighing three times and determining its volume. Each sample’s weight result was divided into volume and averaged to recognize its density.

#### 4.1.8. Equilibrium Swelling Ratio

Each substrate was weighed, then thermostated at 25 °C in the solvent, i.e., water, rapeseed oil, and olive oil, for 24 h with continuous shaking (150 rpm) performed by Promax 1020 (Heidolph, Schwabach, Germany) and reweighed after the procedure. The equilibrium swelling ratio (ESR) was designated by the percent of dividing wet substrate weight by dry substrate weight. Results were replicated three times and averaged.

### 4.2. Biomass of Hairy Roots

In cultures on biodegradable constructs, the *Rindera graeca* hairy root line, originally established and developed by Sykłowska-Baranek et al. [35] via biotechnological methods, was used. The 250 cm^3^ Erlenmeyer flasks containing 50 cm^3^ of hormone-free DCR medium (developed by Gupta and Durzan, 1985 [50]) were utilized to maintain the *R. graeca* roots. Hairy roots containing flasks were agitated on an oscillatory shaker (INFORS AG, Bottmingen-Basel, Switzerland) at 105 rpm and 24 °C in dark conditions. Routine subculturing of *R. graeca* roots was performed every four weeks.

### 4.3. Cultures of Hairy Roots on Biodegradable Constructs

Culture without biodegradable constructs was considered as the reference system.

In the case of cultures supported with biodegradable constructs, round plates of poly(glycerol sebacate) (PGS, φ = 4 cm, 0.3 cm of thickness, 3.4 g) and poly(lactic acid) (PLA, φ = 4 cm, 0.05 cm of thickness, 0.1 g), were separately placed in 250 cm^3^ Erlenmeyer flasks containing 50 cm^3^ of hormone-free DCR medium—two independent culture systems were established. Next, 0.5 g of 28-day *R. graeca* hairy roots (as an inoculum) was poured onto the upper surface of the tested materials. All culture systems were continuously incubated at 24 °C in dark conditions for 28 days on the oscillatory shaker at 105 rpm.

After 28 days, *R. graeca* roots were harvested from culture systems, and fresh biomass weight analysis was conducted. Harvested hairy roots, culture medium (filtered by 0.2 µm syringe filter), and all the materials were carefully separated, quantitatively collected, and individually stored at −20 °C. Before extraction procedures, all collected biomaterials and the hairy roots biomass were lyophilized (Christ ALPHA 1-4 LSC, Christ, Osterode am Harz, Germany). For secondary metabolite extraction from a not-treated culture medium, lyophilized and micronized hairy root biomass, n-hexane (Sigma-Aldrich, Burlington, MA, USA), was applied. An extraction with HPLC-grade methanol (Sigma-Aldrich, Burlington, MA, USA) was performed for the metabolites accumulated in PGS and PLA. All samples were sonicated (Sonorex, Bandelin, Berlin, Germany) to increase the extraction efficiency. Extraction procedures were repeated until the solvent color definitely faded. A more detailed procedure applied for secondary metabolite extraction is available in the previous reports [47].

### 4.4. HPLC Analysis of Extracts

The reversed-phase chromatographic (RP-HPLC) technique and further analysis were performed using the Dionex 3000 HPLC system (by Dionex, a brand of Thermo Scientific, Sunnyvale, CA, USA) coupled with an automated sample injector (ASI-100) and UVDAD 340S UV-Vis diode-array detector. The RP-HPLC analysis was carried out under the following conditions: eluent (gradient)-acetonitrile (60–80%, Sigma Aldrich, Burlington, MA, USA)/0.04 M ortho-phosphoric acid (40–20%, Sigma Aldrich, Burlington, MA, USA), the flow rate of eluent 1.5 cm^3^ min^−1^, EC Nucleosil 120-7 ODS packed column (250 × 4.6 mm, 7 µm particles, 120 Å pores, Macherey-Nagel, Düren, Germany) according to the previously published procedure [8]. The absorbance of eluent was monitored at 215 (as reference wavelength), 237, 350, and 436 nm. According to the external standard methodology, the deoxyshikonin chromatography standards with confirmed identity were used for the peak identification.

### 4.5. Biomass Growth Kinetics

The *R. graeca* hairy roots proliferation was quantitatively described by the values of fresh biomass (FB_28d_) increase, which were determined based on the following equation:

FB_28d_ = m_28d_ · m_0d_^−1^ (−), (1)

where m_28d_ is the fresh biomass weight on the 28th day of culture, and m_0d_ is the biomass inoculum weight.

The yield of deoxyshikonin production per dry biomass mass (Y_P/X_), i.e., 1.0 g of dry biomass values, was calculated from the following equation:

Y_P/X_ = m_n_ · DB_28d_^−1^ (µg g_DW_^−1^), (2)

where m_n_ is the deoxyshikonin weight produced in the culture system, and DB_28d_ is the dry biomass weight on the 28th day of culture.

The values of specific growth rate (µ), which described the growth rate of *R. graeca* roots in studied systems, were determined according to the following equation:

µ = (ln m_28d_ − ln m_0d_) · (Δt)^−1^ (h^−1^), (3)

where Δt is the time of culture.

### 4.6. Statistical Analysis

One-way analysis of variance (ANOVA) with Tukey’s post hoc test was used to designate the statistical difference between the root biomass growth values. In this case, the Shapiro–Wilk and Bartlett’s tests were applied for normal distribution and variance homogeneity, respectively, showing fulfillment of the ANOVA test conditions for all investigated samples. For the rest of the statistical analysis, student’s t test was applied. For all experiments, *p* ≤ 0.05 was considered significant.

## 5. Conclusions

The successful application of constructs made of two polymerized organic-ester-based biomaterials with various chemical properties, i.e., PGS and PLA, for the immobilization of *R. graeca* hairy roots and in situ deoxyshikonin extraction were investigated and widely discussed. The PLA-based constructs promoted the proliferation of hairy roots and the fresh biomass increase, which was over two times higher than in the reference culture non-supported with any type of construct. In our opinion, this result may be caused by PLA scaffolds’ properties. The high porosity, SFE, ERS, and low density of PLA-based constructs provide a suitable environment for *R. graeca* hairy root proliferation. The low density of PLA scaffolds caused the elevation of roots to the surface of the aqueous phase and increased the availability of oxygen, which may be the main factor that promotes hairy root biomass proliferation. In the case of applied PGS-based constructs, the growth of hairy roots was relatively low, but the productivity of deoxyshikonin reached the level of 1983 µg g_DW_^−1^ in contrast to the lack of deoxyshikonin detection in both the reference system and the culture system supported with PLA-based constructs. We suppose that the phenomena in the PGS-based constructs culture system were caused by stress factors, which do not occur in other culture systems, i.e., limited access to the water phase and nutrient deprivation. These hypothetical stress factors, as well as products of the degradation of PGS constructs, could influence the secondary metabolism and inhibit the growth of *R. graeca* hairy roots. However, further detailed studies are required to elucidate the mechanism of deoxyshikonin biosynthesis induction in *R. graeca* hairy roots cultivated on polymerized organic-ester-based biomaterials that could lead to enhanced productivity of the proposed culture systems.

In summary, the physiological and biochemical response of *R. graeca* hairy roots (i.e., roots growth and secondary metabolite production) differed depending on the type of applied scaffold. The PLA scaffold promoted the proliferation of transgenic roots and, in further studies, can be applied for accelerated plant biomass production and rapid inoculum preparation for large-scale cultures. In the case of the PGS scaffold, which resulted in the high production of secondary plant metabolites, the main future application can be intensification or event induction of the secondary metabolites production in various plant species. Moreover, the blend of PLA and PGA as one scaffold for the simultaneous production of secondary plant metabolites and plant biomass growth promotion will be applied in future studies.

## Figures and Tables

**Table 1:** The values of SFE and density estimated for studied constructs made of PGS and PLA.

Material	SFE (mJ m^−2^)		Density (g cm^−3^)	
	Value	*σ*_SD_	Value	*σ*_SD_
PGS	1.74	2.93 × 10^−2^	1.121	4.54 × 10^−2^
PLA	15.2	27.4 × 10^−2^	0.127	0.77 × 10^−2^

**Table 2:** Values of m_n_ and Y_P/X_ characterizing the production of deoxyshikonin derivative by *R. graeca* hairy roots in comprised in vitro culture systems: the reference system without any scaffold and two systems supported with organic ester-based scaffolds made of PGS and PLA.

Culture System	*m_n_* (µg)		*Y*_P/X_ (µg g_DW_^−1^)	
	Value	*σ*_SD_	Value	*σ*_SD_
Reference system	0.0	0.00	0.0	0.00
PGS	157.4	37.75	1983	404.3
PLA	0.0	0.00	0.0	0.00

**Table 3:** Previously published data on materials applied for the immobilization of hairy roots.

Material/ Construct	Metabolite	Plant Species	V_L_ (dm^3^)	Y_P/X_ (µg g_DW_^−1^)	FB_28d_ (−)	References
Amberlite XAD-4	Diterpenoids	*Salvia miltiorrhiza*	0.05	550	n/a	[42]
Amberlite XAD-7	Thiophenes	*Tagetes patula*	0.05	294	3.2	[43]
DIAION HP-20 PS resin	Stilbenes	*Arachis hypogaea*	0.05	11,976	n/a	[44]
MTMS aerogel	Naphthoquinones	*Rindera graeca*	0.05	636	6.07	[8]
PGS			0.05	1983	1.20	current study
PLA			0.05	0	8.85	
PP (disk)		*Plumbago rosea*	1	1500	2.8	[12]
PP (mesh)	Tetranortriterpenoids	*Azadirachta indica*	1	2100	3.16	[13]
PUF			1	6400	5.10	[10]
PUF	Alkaloids	*Catharanthus roseus*	3	1130	3.17	[11]
	Naphthoquinones	*Rindera graeca*	0.05	653	2.18	[8]
X-5 PS resin	Diterpenoids	*Salvia miltiorrhiza*	0.05	560	n/a	[42]

## Data Availability

The data presented in this study are available on request from the corresponding author.
